# Retrievable Inferior Vena Cava Filters for Venous Thromboembolism

**DOI:** 10.5402/2013/959452

**Published:** 2013-04-22

**Authors:** Han Ni, Lei Lei Win

**Affiliations:** ^1^Internal Medicine, Faculty of Medicine, SEGi University, No. 9 Jalan Teknologi, Taman Sains Selangor, Kota Damansara, PJU 5, 47810 Petaling Jaya, Selangor, Malaysia; ^2^Paediatrics, Faculty of Medicine, SEGi University, No. 9 Jalan Teknologi, Taman Sains Selangor, Kota Damansara, PJU 5, 47810 Petaling Jaya, Selangor, Malaysia

## Abstract

Inferior vena cava (IVC) filters are used as an alternative to anticoagulants for prevention of fatal pulmonary embolism (PE) in venous thromboembolic disorders. Retrievable IVC filters have become an increasingly attractive option due to the long-term risks of permanent filter placement. These devices are shown to be technically feasible in insertion and retrieval percutaneously while providing protection from PE. Nevertheless, there are complications and failed retrievals with these retrievable filters. The aim of the paper is to review the retrievable filters and their efficacy, safety, and retrievability.

## 1. Introduction

Venous thromboembolism (VTE), including deep vein thrombosis (DVT) and pulmonary embolism (PE), is a cause of significant morbidity and mortality in both hospitalized and nonhospitalized patients. Approximately 400,000 to 650,000 patients develop PE annually with 50,000 to 240,000 deaths in the United States [1]. Standard therapy is parenteral anticoagulants (full-dose unfractionated heparin, low-molecular-weight heparin, or fondaparinux) followed by oral vitamin K antagonists (warfarin). However, in cases of contraindications to anticoagulants, bleeding complications, or recurrent VTE despite optimal anticoagulation, interruption of inferior vena cava (IVC) with a filter is necessary to prevent life-threatening PE [2].

## 2. Types of IVC Filters

The characteristics of an ideal IVC filter include high filtering efficiency without impedance of flow, secure fixation within IVC, rapid percutaneous insertion (small calibre, amenable to repositioning), MRI compatibility, low cost, and retrievability. Moreover, the ideal filter should be made of nonthrombogenic, biocompatible long-lasting material [3, 4]. Nevertheless, none of the currently available IVC filters meet all these criteria.

IVC filters are implanted as permanent or nonpermanent. Mobin-Uddin filter was first introduced in 1967. However, due to high incidence of thrombosis and occlusion, Greenfield filter quickly became the preferred choice, which was first described in 1973 [5–7]. This Greenfield stainless steel filter and another permanent Bird's Nest filter are MRI incompatible. Other permanent IVC filters available are Simon Nitinol, TrapEase, and VenaTech, which are all MRI compatible [3, 8]. Subsequent studies demonstrated the increased incidence of complications associated with permanent IVC filters [9]. One of the significant long-term risks of permanent filters is thrombotic occlusion of the IVC, which is seen in 6% to 30% of cases; other important complications include vena cava perforation, filter dislocation, migration, rupture, recurrent venous thromboembolism, thrombophlebitis, and venous stasis disease [2, 10, 11].

To reduce long-term complications related with permanent filters, nonpermanent IVC filters are being developed, which were first approved by the US Food and Drug Administration (FDA) in 2003 [12, 13]. The optional IVC filters can be either removed from patients once their risk of thromboembolic disease has reduced (retrievable filters) or altered in some means to cease functioning as a filter while it remains in the IVC (convertible filters) [2, 14].

**Figure 1:**
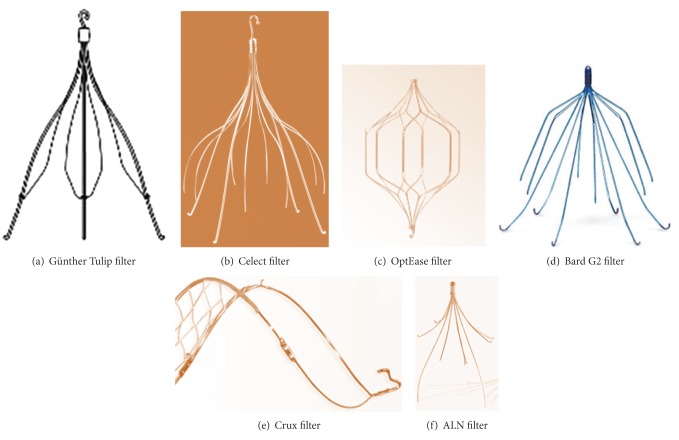
Retrievable IVC filters: (a) Günther Tulip filter, (b) Celect filter, (c) OptEase filter, (d) Bard G2 filter, (e) Crux filter, and (f) ALN filter.

The FDA-approved retrievable IVC filters are Günther Tulip and Celect filters (Cook Medical), OptEase filter (Cordis Endovascular), and G2 and G2 Express filters (Bard Peripheral Vascular). Crux vena cava filter (VCF) is the recently FDA approved bidirectional retrievable filter (Crux Biomedical). ALN filter with hook has also achieved FDA approval recently (ALN Implants Chirurgicaux, France) (Figure 1).

### 2.1. Günther Tulip Filter

The Günther Tulip filter was first developed in 1992 in Europe and has been available since 2000 in the United States. It can be inserted either through femoral or jugular approach using 8.5F introducer sheath. This filter can be used for maximum caval size of 30 mm. Hooks at the caudal end of the legs anchor the device to the wall of IVC while the rounded tip hook at the cranial apex is for snare retrieval [2, 8, 15]. A separate Günther retrieval kit or any endovascular snare is used to retrieve the filter through internal jugular vein [2]. The hook at the apex of the filter is snared and a 9F sheath is used to collapse the filter. After the filter has been completely sheathed, the sheath/filter combination is withdrawn from the patient. Though it is mainly retrieved from internal jugular approach, van Ha et al. have reported a case where Günther Tulip filter was removed by a new technique through the femoral approach due to the occlusion of internal jugular and subclavian veins from previous central catheter placement [16].

### 2.2. Celect Filter

The Celect filter is the second generation optional IVC filter from Cook Medical after the Tulip filter with redesigned legs for secure, atraumatic caval fixation. Moreover, secondary strut design centers the filter with minimal ingrowth. Insertion is either through jugular or femoral veins, but retrieval of the filter can be performed only by the jugular approach.

### 2.3. OptEase Filter

The OptEase retrievable filter is the only filter retrievable from a femoral vein approach. Insertion is from either jugular or femoral approach using a 6F introducer system. It is recommended for use in patients with a caval size of 30 mm or less. The caudal apex of the filter is formed into a T-shaped retrieval hook for retrieval with an endovascular snare device inserted through a 7F to 12F sheath via femoral approach. The snare engages the caudal retrieval hook, and the sheath is then advanced over the filter. The filter subsequently collapses and is withdrawn through the sheath [2, 8, 17].

### 2.4. Bard G2 Filter

The second generation Bard G2 filter consists of two levels for filtration of emboli. The legs provide the lower level and the arms provide the upper level of filtration. It is used in the inferior vena cava (IVC) with a diameter less than or equal to 28 mm. Insertion is through femoral or jugular/subclavian approach using 7F or 10F separate delivery systems. For retrieval, Bard Recovery Cone Removal System was advanced via jugular approach and docked with the filter tip so that the filter could be retracted into the sheath and removed. In a single-center, retrospective, cross-sectional study conducted from 2004 until 2009, Bard Recovery (first generation) and Bard G2 filters were found to be associated with high prevalence of fracture and embolization, with potentially life-threatening sequelae. Six of 52 Bard G2 filters fractured (12%) and in 2 of these 6 cases, the patients had asymptomatic end-organ fragment embolization [18]. In another retrospective study at a single institution from 2004 to 2010 among patients with Bard Recovery, G2, and G2 Express filters presenting for filter removal, overall fracture rate of 12% was reported (63 of 548 patients). However, clinically significant complications of IVC filter fracture were uncommon with no reported immediate clinical sequelae due to embolization of fracture components [19].

### 2.5. Crux Vena Cava Filter

The Crux filter is designed to facilitate bidirectional retrieval through either femoral or jugular veins. It is a self-expanding filter where wireforms are composed of two opposing self-expanding nitinol spiral elements connected at each end with nitinol crimps. One end of each wireform is formed into a sinusoidal shaped retrieval tail to help in retrieval of the filter using a snare. Each retrieval tail has a plasma ball and a radioopaque tantalum marker band to facilitate visualization. There are five tissue anchors attached to the wireforms elements with nitinol tubing for fixation with minimal perforation. The filter is designed for IVC diameters of 17 to 28 mm and inserted using a 9 Fr catheter.

### 2.6. ALN Filter

The ALN filter with hook has the same features as the optional ALN IVC filter. It consists of six short legs that adhere to the IVC wall, and three long legs to ensure the correct central positioning along the main axis of IVC. It is characterized by low thrombogenicity and less chance of occlusion, because of the lesser caval section it occupies and the low amount of metal used. Furthermore, the absence of welding points results in an excellent corrosion resistance. This filter can be placed from femoral, brachial, or jugular approach but can be retrieved only from jugular approach [20].

## 3. Indications and Contraindications

The indications for implantation of permanent filters are applicable for all retrievable filters. Proven acute PE or proximal DVT with contraindications to anticoagulation therapy and recurrent VTE despite adequate anticoagulation are absolute indications for placing IVC filters [3, 21]. The American College of Chest Physicians guidelines do not recommend the use of IVC filters as an additional protective therapy in proven VTE cases being treated with anticoagulation [21]. Relative indications for IVC filters include proven VTE with limited cardiopulmonary reserve, poor compliance with anticoagulation, high risk of complications of anticoagulant therapy, iliocaval DVT, large free-floating proximal DVT, thrombolysis for iliocaval DVT, and massive PE treated with thrombolysis/thrombectomy. Prophylactic indications for IVC filters include trauma, surgery, and medical conditions with high risk of VTE [4]. Nevertheless, relative and prophylactic indications are not yet recommended by the existing guidelines [21].

No accessible route to vena cava and no location available in vena cava are contraindications to filter placement [4, 22].

## 4. Placement and Retrieval

Placement and removal of retrievable IVC filters can be performed safely with a high technical success rate [23–25].

Imaging has a role in filter placement and retrieval. A recent study reported that the intravascular ultrasound-guided IVC filter placement using a single venous puncture technique is technically feasible and safe compared to double venous puncture technique [26].

Retrievable IVC filters are deployed in the same manner as permanent filters but they can be retrieved percutaneously when IVC interruption is no longer needed. The period from implantation of filter to the safe retrieval is termed as the window of retrievability. Desirable characteristics for retrievable IVC filters include long window of retrievability, easy retrievability from both jugular and femoral approaches, and feasibility to be left in place as a permanent filter if a patient requires continued filtration [17].

Technically, removal is more difficult than placement [42]. Retrieval is done percutaneously using a commercially available snare or the standard recovery systems depending on the type of filter. Either jugular or femoral approach is applied for retrieval of most of the IVC filters until the recent development of new Crux filter, which can be retrieved from both directions.

Though prolonged dwell time does not increase the complication rate [43], risk of retrieval failure increases with longer duration of filter placement [41]. The reported technical and clinical success rate of filter retrieval is reported to be 100% if removed within 14 days [2, 24]. Statistical estimates revealed the probability of successful device retrieval more than 94% at 12 weeks and more than 67% at 26 weeks for Günther Tulip filter [27] and 100% at 50 weeks and more than 74% at 55 weeks for Celect filter [30].

If there is a need to prolong temporary IVC filtration beyond the recommended period of 14 days, percutaneous repositioning of the filter via internal jugular approach to a different location within the IVC before definitive device removal can be helpful [29]. Successful filter removal was documented up to 3 years after placement [42]. Modified retrieval techniques along with adjunctive necessary endovascular maneuvers help in removal of adherent IVC filters implanted for up to 5 years [44].

## 5. Efficacy, Safety, and Retrievability

Currently available evidences suggest that IVC filters are largely effective and safe when used appropriately [28, 45]. Prophylactic temporary IVC filter placement is simple and safe, effectively prevents devastating PE, and serves as a “bridge” to anticoagulation [23, 38]. IVC filters are shown to reduce the risk of pulmonary embolism at 8 years; however, there is an increased risk of recurrent DVT and there is an increased risk of recurrent DVT with no effect on survival [4, 9]. IVC filters are also reported to be effective in preventing fatal PE in trauma patients with DVT in perioperative period [39]. Thus, prophylactic use of IVC filters has become a standard practice at some trauma centers though it is not recommended by the existing guidelines [33].

Insertion of temporary retrievable IVC filters for venous thromboembolic disease has been performed more widely in the recent years. However, few “retrievable filters” are actually removed, with most published series documenting a retrieval rate between 20% and 50% [40] with mean retrieval rate of 34% [46]. Suboptimal IVC filter retrieval rate can lead to complications associated with long-term placement [47]. Low retrieval rates are mainly due to loss of followup. In a retrospective analysis of medical records in a center in Australia, it was found that 61% of patients who have undergone retrievable IVC filter insertion received no clinical followup. Factors associated with loss of followup include lack of haematology outpatient clinic review after discharge (*P* < 0.01), absence of documentation for retrieval plan (*P* < 0.01), and age greater than 50 years (*P* < 0.01) [48].

Various measures have been experimented to achieve higher retrieval rate. In a study, the patients with retrievable IVC filters were enrolled into a dedicated filter registry. Initial contacts with patients were done by telephone. If unsuccessful with phone contact, then family members, rehabilitation facility, and social work were all contacted to obtain the most recent contact phone number and address. A letter was also sent to the patient with detailed follow-up visit instructions. Finally, a certified letter was delivered to the last known address if other measures failed. With this strategy of improved care, higher retrieval rate of 59% was achieved [40]. Higher retrieval rate by dedicated tracking of patients has also been shown in another study where the tracked patients had significantly higher rates of filter retrieval (60% versus 30%, *P* = 0.02) and filter retrieval attempts (70% versus 30%, *P* = 0.002) compared to those without dedicated tracking. The tracking was also associated with significantly less chance of lost of followup (5% versus 65%, *P* < 0.0001) [49].

Despite low rate of IVC filters removed in relation to the number inserted, technical success rate is high for those removed, with substantially low retrieval failure rate. Failed retrieval may be due to attachment of the filter to the IVC wall as a result of excessive tissue growth, extreme filter tilting, or extensive filter thrombus (Table 1). Retrieval failure was associated with patient age more than 80 years (odds ratio (OR) 0.056, *P* < 0.0001), presence of malignancy (OR 0.303, *P* = 0.003), and time interval more than 90 days between implantation and attempted retrieval (OR 19.8, *P* = 0.009) [41]. Technical failure accounts for 5.8% of failed retrievals [32] and is directly proportional to filter tilt >15° [42].

**Figure 2:**
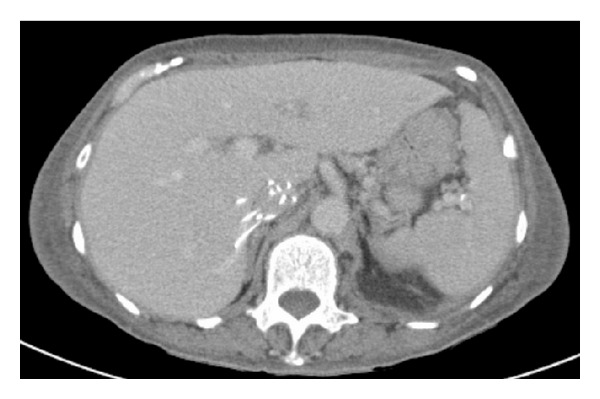
Preretrieval CT image showing obliquely oriented filter with perforation of IVC by the filter struts both medially and anteriorly.

Preretrieval CT appearance can be helpful in predicting complications during retrieval (Figure 2). In a recently published study, mediolateral and anteroposterior tilt angle, degree of perforation, and dwell time were higher in the complicated group compared to the noncomplicated retrieval group (*P* < 0.01). Odds of complicated retrieval were increased 129-fold with CT appearance of tip embedding (*P* < 0.0001) and 33-fold with a tilt angle of more than 15° in any direction (*P* < 0.0001). Perforation and dwell time increased the risk of complicated retrieval by 10.7 (*P* < 0.0001) and 2.3 (*P* < 0.05) times, respectively. Distance of filter from renal veins had no association. Thus, CT imaging before the retrieval procedure is advisable for detection of high risk factors to modify retrieval approach or to refer to a tertiary center if necessary [50].

## 6. Complications

**Figure 3:**
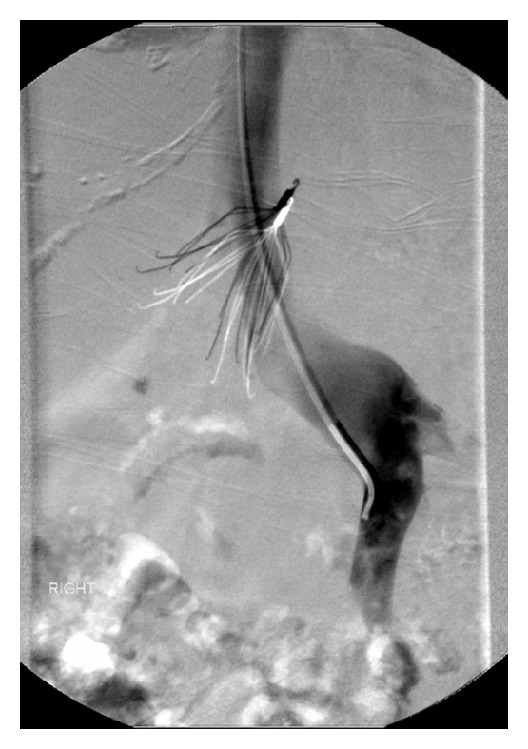
Migration of the filter with penetration of IVC.

The important complications include filter occlusion and IVC thrombosis (6%–30%), recurrence of lower limb DVT and postthrombotic syndrome (5%–70%), IVC perforation either symptomatic or radiological extension of filter components more than 3 mm outside IVC wall (9%–24%), filter migration (3%–69%) (Figure 3), insertion site thrombosis (2%–28%) and complications from insertion (4%–11%) [2].

Bard filters are reported to be associated with high rate of strut fracture (16%) and fragment embolization (25%); of interest, in five of seven cases, at least one fragment embolizes to the heart (71%). Three patients experienced life-threatening ventricular tachycardia and/or cardiac tamponade, of which one had sudden death at home [18].

High penetration rate is seen with Celect IVC filters, including penetrations that were symptomatic or involved adjacent structures. Penetration correlates with indwelling time, suggesting prompt filter removal as soon as the indication for PE protection is alleviated [32]. Penetration may lead to injury of adjacent bowel, kidney, pancreas, and aorta with risk of pseudoaneurysm formation [51].

## 7. Special Situations

### 7.1. Suprarenal Placement

Although most of the filters are placed in infrarenal portion of IVC, there are few exceptions where they are placed above the renal veins. Indications for suprarenal placement of IVC filters include IVC thrombus, intrinsic and/or extrinsic narrowing of the infrarenal IVC, renal and/or gonadal vein thrombus, congenital IVC anomalies, pelvic mass, and pregnancy [52].

In a follow-up study of 22 patients with suprarenal IVC filter placement, the procedure was proved to be safe, with no evidence of permanent renal impairment after the placement. Filter migration was the most frequent complication, but no clinically significant sequelae were noted in these patients. The increased chance of filter migration in suprarenal placement might be attributable to the larger diameter of the suprarenal IVC as well as its variability due to venous return, blood volume, and respiratory cycle [53].

A similar study on implantation of IVC filters in suprarenal position in thirteen patients with renal cell carcinoma (RCC) and renal vein thrombosis with or without extension into IVC reported 100% feasibility in both insertion and removal. All filters were correctly deployed in the suprarenal tract of the IVC with no peri- or postprocedural complications. There was no evidence of PE in the 30 days after the procedure. All suprarenal IVC filters were removed 30 to 60 days after surgical resection of RCC [54].

### 7.2. Children and Elderly

The use of IVC filters in children is not well reported as in adults, with long-term studies lacking. In a study on three young children (two to three years of age) over a 14-month period, IVC filter was placed via internal jugular vein in two and femoral vein in one. The filters were deployed successfully in all three children and retrieved in two. Removal was not attempted in one child who was on palliative care. There were no complications during placement, dwelling, or retrieval [55].

Another retrospective review mentioned 100% success in placement without complications in thirty-five children (mean age: 15.5 years). Filter retrieval was successful in 15 of 19 attempted (79%) at mean duration of 42 days. Retrieval failure in four children is due to endothelialization of filter. Persistence of filters was associated with acceptable complication rate on followup in this population [56].

Though a study has shown that age more than 80 year is associated with retrieval failure [41], a retrospective review of retrievable IVC filters in elderly population of more than 65 years of age reported that the filters are safe and effective. Technical success rates for optional filter placement and retrieval were 98.1% (53 of 54) and 55.6% (30 of 54), respectively. Age alone is not a poor predictor of possible filter removal. There was no incidence of PE after optional filter placement. Therefore, appropriate patient selection and intensive followup in elderly can result in retrieval rates comparable with younger population [57].

## 8. Conclusion

With the advent of modern interventional radiology, retrievable IVC filters are used with increasing frequency. Studies have shown that these filters are safe and effective in thromboembolic disease; however, most of these studies are unrandomized with short duration followup. Furthermore, the retrieval rate is suboptimal in most of the studies though technical success rate is high. Thus, it is also important to practice better care to ensure strict attendance to followup for timely retrieval. Dedicated tracking system in a systematic registry and properly documented follow-up plan for those with retrievable IVC filters may prevent unnecessary continuation of the filters by ensuring prompt attempted retrieval once the indication has been removed. With these measures, untoward consequences of chronic implantation of filters can be prevented.

## Figures and Tables

**Table 1:** Results of retrievable IVC filters studies.

Study	Type of filter	Number of filters placed	Number of filters removed	Dwelling time of filters mean; range (days)	Successful retrieval rate	Reasons for failed retrieval
Smouse et al. [27]	Günther Tulip	554	275	58.9; 3–494	248 of 275 (90.2%)	Improper hook orientation (*n* = 10) and excessive tissue growth (*n* = 16)
Terhaar et al. [24]	Günther Tulip	53	19	34; 7–126	16 of 19 (84%)	Extensive filter thrombus (*n* = 2) and attachment to the wall (*n* = 1)
Looby et al. [28]	Gunther Tulip	147	45	33.6	36 of 45 (80%)	Attachment to the IVC wall (*n* = 5), extreme filter tilt (*n* = 2), and extensive filter thrombus (*n* = 2)
de Gregorio et al. [29]	Günther Tulip	88	70	13 (no repositioning *n* = 46) 34.8 (repositioning *n* = 23)	70 of 70 (100%)	—
Ray et al. [23]	Günther Tulip (143) Recovery filter (54)	197	94	11; 1–139 (Günther Tulip) 28; 6–117 (Recovery)	80 of 94 (85.1%)	Extensive filter thrombus (*n* = 7), filter embedded in IVC wall, and tilted filter (*n* = 7)
van Ha et al. [25]	Günther Tulip (44) Recovery filter(53)	97	29	226; 2–1217	28 of 29 (96.6%) (14 of 15 (Recovery) 14 of 14 (Günther Tulip))	Large filter clot (*n* = 1)
Lyon et al. [30]	Celect	95	58	179; 5–466	56 of 58 (96.6%)	Tilting (*n* = 1) and excessive tissue growth (*n* = 1)
Sangwaiya et al. [31]	Celect	73	14	84 (median)	14 of 14 (100%)	—
Zhou et al. [32]	Celect	620	120	158.1; 2–518	106 of 120 (88.3%)	Filter embedment (*n* = 6), caval occlusion (*n* = 3), retained thrombus (*n* = 2), large floating IVC thrombus (*n* = 2), and tilt > 15° (*n* = 1)
Sebunya et al. [33]	Recovery G2 (88%) Celect (11%) Unspecified (1%)	78	40	100; 12–349	36 of 40 (90%)	—
Oliva et al. [34]	OptEase	27	21	11.1; 5–14	21 of 21 (100%)	—
Rosenthal et al. [35]	OptEase	40	40	16; 3–48	40 of 40 (100%)	—
Onat et al. [36]	OptEase	228	124	11; 4–23	115 of 124 (91%)	—
Kalva et al. [37]	OptEase	71	14	9; 5–21	12 of 14 (85%)	—
Rosenthal et al. [38]	Günther-Tulip (49), Recovery G2 (41), and OptEase (37)	127	66	—	66 of 66 (100%)	—
Shao et al. [39]	Various	399	389	22.8; 7–60	389 of 389 (100%)	—
Rogers et al. [40]	Various	420	160	—	94 of 160 (59%)	—
Geisbusch et al. [41]	Various	200	91	—	85 of 91 (93.4%)	—
Lagana et al. [42]	ALN	201	26	—	25 of 26 (96.2%)	—

## Abbreviations

VTE: Venous thromboembolism
DVT: Deep vein thrombosis
PE: Pulmonary embolism
IVC: Inferior vena cava
MRI: Magnetic resonance imaging
FDA: Food and Drug Administration
CT: Computed tomography
RCC: Renal cell carcinoma
VCF: Vena cava filter.
